# Prevalence of Anxiety Symptoms and Their Association With Loss Experience in a Large Cohort Sample of the Oldest-Old. Results of the AgeCoDe/AgeQualiDe Study

**DOI:** 10.3389/fpsyt.2019.00285

**Published:** 2019-05-08

**Authors:** Franziska D. Welzel, Janine Stein, Susanne Röhr, Angela Fuchs, Michael Pentzek, Edelgard Mösch, Horst Bickel, Siegfried Weyerer, Jochen Werle, Birgitt Wiese, Anke Oey, André Hajek, Hans-Helmut König, Kathrin Heser, Luca Keineidam, Hendrik van den Bussche, Carolin van der Leeden, Wolfgang Maier, Martin Scherer, Michael Wagner, Steffi G. Riedel-Heller

**Affiliations:** ^1^Institute of Social Medicine, Occupational Health and Public Health (ISAP), Medical Faculty, University of Leipzig, Leipzig, Germany; ^2^Institute of General Practice, Medical Faculty, Heinrich-Heine-University Düsseldorf, Düsseldorf, Germany; ^3^Department of Psychiatry, Technical University of Munich, Munich, Germany; ^4^Central Institute of Mental Health, Medical Faculty Mannheim/Heidelberg University, Mannheim, Germany; ^5^Institute for General Practice, Hannover Medical School, Hannover, Germany; ^6^Department of Health Economics and Health Services Research, University Medical Centre Hamburg-Eppendorf, Hamburg, Germany; ^7^Department for Neurodegenerative Diseases and Geriatric Psychiatry, University Hospital Bonn, Bonn, Germany; ^8^German Center for Neurodegenerative Diseases, Bonn, Germany; ^9^Department of Primary Medical Care, Center for Psychosocial Medicine, University Medical Center Hamburg-Eppendorf, Hamburg, Germany

**Keywords:** prevalence, anxiety, loss, old age, primary care

## Abstract

**Background:** Anxiety in adults is a common mental health problem. However, studies on anxiety in the oldest-old are lacking. We sought to identify the age- and gender-specific prevalence of anxiety symptoms in a large sample of general practice patients. Furthermore, we investigated relevant associations of anxiety specifically with respect to recent experience of loss.

**Methods:** Based on the German Study on Ageing, Cognition and Dementia in general practice patients, a sample of 897 patients aged 82 years and older was assessed. Anxiety was assessed using the short form of the Geriatric Anxiety Inventory (GAI-SF). For the assessment of loss, patients were asked whether there were cases of death in their closer social environment since the last assessment. Descriptive and logistic regression analyses were run.

**Results:** Of the oldest-old individuals (aged 82+ years, mean age: 86.8), 14.5% (95% CI 12.4–16.8) suffered from anxiety symptoms. Highest prevalence rates were found for 82- to 85-year-old women (17.2%, 95% CI 12.6–22.1) and for 86- to 90-year-old patients (both sexes) in general (15.9%, 95% CI 12.6–19.2). Older individuals who experienced cases of death in their close social environment within the last 18 months had almost twice the odds [odds ratio (OR) 1.91, 95% confidence interval (CI) 1.15–3.17] of reporting anxiety compared to those without a recent loss. As expected, depression and impaired cognitive status were associated with the presence of anxiety symptoms. No relation was found between social network, gender, age, frailty, or physical illness and anxiety in regression analysis.

**Conclusions:** This study provides for the first time age- and gender-specific prevalence rates of anxiety symptoms and associated risk factors among a large population-based sample of oldest-old primary care attenders. Anxiety is highly prevalent in individuals aged 82 years and older. Depression, impaired cognitive status, and recent experience of loss are associated with late-life anxiety. Our findings support the idea that recent experience of loss should be taken seriously in the context of clinical practice with respect to diagnosing and treating anxiety in old age.

## Introduction

Understanding mental health problems in old age becomes a growing need alongside with the growth of older people among the world’s population. According to the German Federal Statistical Office, one in three people of the German population will be 65 years or older by 2060 (1). While the proportion of individuals in old age has increased in European countries over the past decade, research on mental health issues extended their scope of interest to late life. Anxiety disorders have been reported to be one of the most common mental health problems in older people (2). Although there are some results on anxiety in old age, samples often focused on the so-called “younger” older adults (e.g., 55+ or 65+ years). In a recent cross-sectional multicenter study conducted in Europe and Israel among adults aged 65–84 years, the authors found a 12-month prevalence rate of 17.2% for the presence of any anxiety disorder (3). Other studies reported a rather large variation in the prevalence of anxiety disorders ranging from 1.2% to 14.2% in adults aged 55 years and older (4–9). In general, the prevalence rate of anxiety disorders is reported to be lower among older adults compared to younger adults (3, 10, 11).

Still, older people may be a specific vulnerable group with respect to the development and maintenance of anxiety. The experience of loss and bereavement are frequent negative life events in later life and may pose a significant risk for mental health in old age (12). It has been suggested that bereavement may increase worrying and that worry could hinder the adjustment after bereavement (13). Similarly, psychiatric morbidity including anxiety disorders has been found to be considerably elevated in bereaved spouses in general (14–18). While it has been suggested that older adults may be better prepared for such adverse life events and may have developed better coping mechanisms through life experience (19, 20), research indicates an increase in anxiety symptoms in older adults after bereavement (17, 18).

Previous studies have linked anxiety in later life to female gender, chronic medical or mental illnesses, frailty, cognitive impairment, and the experience of recent adverse life events (9, 21, 22). However, the mechanisms underlying symptoms of anxiety in latest life are not fully understood and may differ in the oldest-old (>80 years) from “younger” people in old age. Especially, adverse life events as the experience of loss may have a pivotal role in the development and maintenance of anxiety at a very old age as personal resources for self-reliance may be less pronounced in this age group. However, to the best of our knowledge, studies analyzing anxiety symptoms in the oldest-old are lacking.

In this study, we therefore sought to identify the age- and gender-specific prevalence of anxiety symptoms in a sample of older (82+) individuals and to investigate risk factors, specifically the possible influence of recent experience of loss, predictive of anxiety in old age. Therefore, it was predicted that anxiety symptoms are highly prevalent in the oldest-old and that the experience of loss is a potential risk factor for anxiety in this age group.

## Materials and Methods

Data were derived from the longitudinal German Study on Ageing, Cognition and Dementia in Primary Care Patients (AgeCoDe) and its follow-up study Needs, health service use, costs and health-related quality of life in a large sample of oldest-old primary care patients (85+) (AgeQualiDe). The AgeCoDe study was conducted as a collaboration of six study centers (Hamburg, Bonn, Düsseldorf, Leipzig, Mannheim, and Munich) and started in 2003–2004 with the baseline assessment. Participating patients were reassessed in follow-ups (FUs) every 18 months until 2013. Based on the AgeCoDe, the AgeQualiDe study continued to assess the same patients with an interval of 10 months until 2017. Participants were recruited through participating general practices (GPs). Each study center included 19–29 GPs. GP patients were recruited based on the following inclusion criteria: a) aged 75+ years, b) absence of dementia, and c) at least one GP contact within the last 12 months. Patients were excluded, if d) GP consultations were home visits only, e) patients lived in a nursing home, f) GPs diagnosed a severe illness that would deem fatal within 3 months, and g) patients were deaf or blind, lacked sufficient proficiency in the German language, or lacked an ability to provide informed consent.

**Figure 1:**
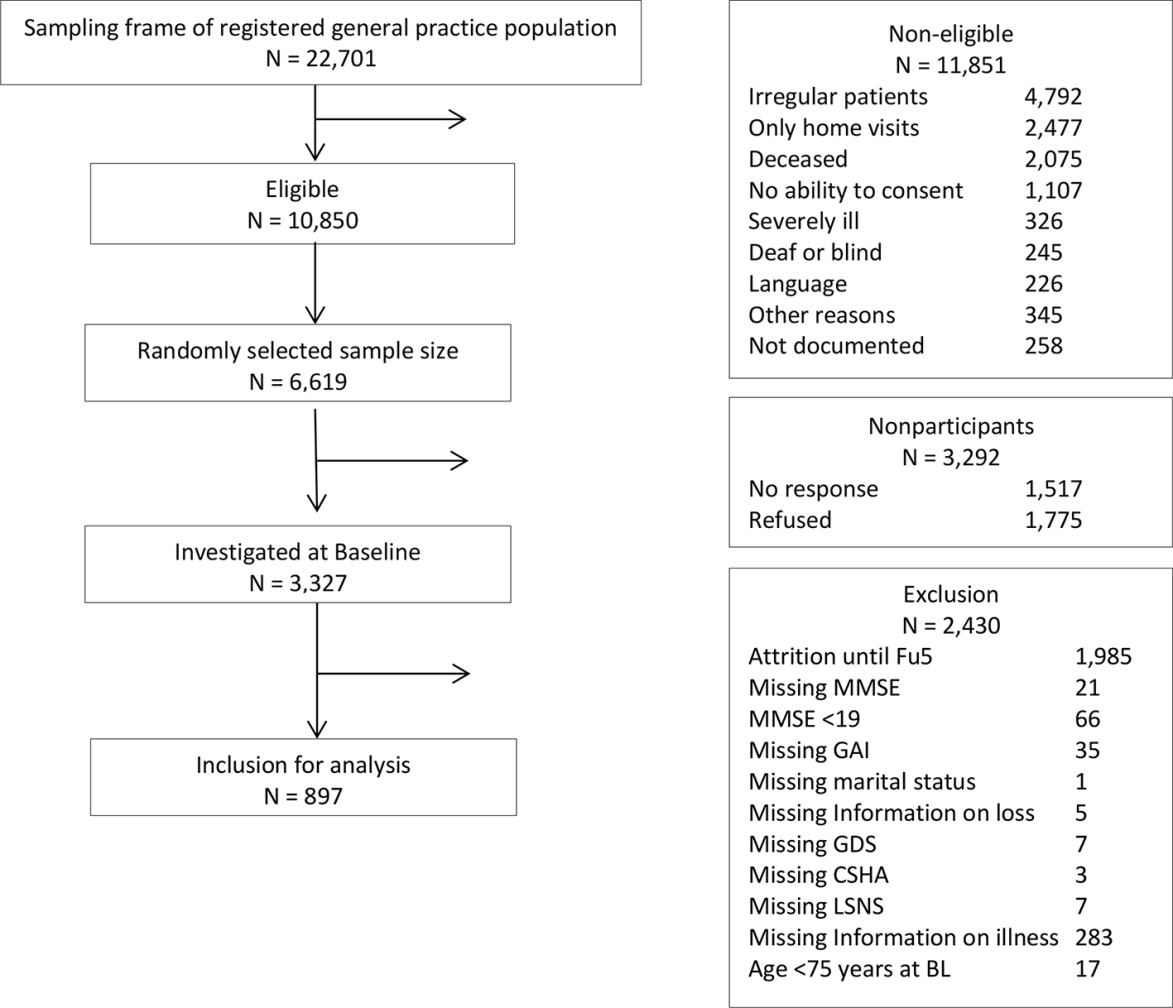
Sample selection flow chart.

Out of a randomly selected sample of N = 6,619 GP patients, a total of N = 3,327 eligible subjects consented to participate and were assessed at baseline through structured clinical interviews. The design of the study has been described in detail elsewhere (23). The present study refers to data from the follow-up 5 (FU5, data assessment 2010–2012) as it includes the assessment of anxiety symptoms in oldest-old first time. A total of N = 897 patients were included for cross-sectional analysis, with N = 2,430 patients being excluded due to study attrition (N = 1,985), incomplete assessments at FU5 (N = 362), or not meeting the inclusion criteria at baseline assessment (N = 17). We further excluded patients with <19 points on the Mini-Mental State Examination (MMSE) to ensure validity of the patients’ short form of the Geriatric Anxiety Inventory (GAI-SF) ratings (N = 66). **Figure 1** provides a detailed overview on the sample selection process.

### Ethics

The ethics committees of all six study centers have approved the study. The study was performed in accordance with the ethical standards of the Declaration of Helsinki (24). Patients and/or their proxies provided written informed consent prior to their study participation.

### Instruments

Data of patients were collected through standardized clinical interviews including standardized tests and assessment instruments conducted by trained interviewers who visited GP patients at home. The standardized interview provided information on a variety of issues. Sociodemographic characteristics included age, gender, and marital status. Educational level was classified as low, medium, or high according to the new Comparative Analysis of Social Mobility in Industrial Nations (CASMIN) educational classification (25).

Anxiety symptoms were assessed *via* the GAI-SF (26). The GAI-SF consists of five items assessing the degree of anxiety symptoms with a yes/no response format and a scale for the sum score ranging from 0 to 5. The GAI-SF contains the following five items: “I worry a lot of the time,” “Little things bother me a lot,” “I think of myself as a worrier,” “I often feel nervous,” and “My own thoughts often make me nervous” (26). The GAI-SF was specifically developed to assess anxiety symptoms in older adults and has been shown to have good psychometric properties [Cronbach’s α = 0.81, (26, 27)]. The GAI-SF has been promoted to be used as a screening instrument for anxiety disorders in older people (27). We identified patients with anxiety with a cutoff by ≥3 (27, 28).

For the assessment of loss, patients were asked whether there were cases of death in their closer social environment within the 18 months since the last assessment. For those patients who had suffered a loss since the last assessment, the degree of relationship and the date of death were assessed for each case of death.

Depressive symptoms were identified using the short version of the Geriatric Depression Scale (GDS) (29). The GDS includes 15 items with a yes/no response scale, and the German version of the GDS has been shown to have good psychometric properties (30). We identified depressive symptoms with a cutoff by ≥6 (30).

The cognitive status of patients was assessed with several instruments including the MMSE (31). The MMSE is a 30-item scale scored 0–30. Patients with a MMSE score of less than 19 points were excluded from further analysis to ensure validity of the patients’ GAI ratings. This cutoff score has been used in previous studies to discriminate patients with suspected severe to moderate dementia and ensure patient ratings (3, 32).

Information on social isolation has been collected using the six-item version of the Lubben Social Network Scale (LSNS-6) (33) scored 0–30, with a cutoff of <12 indicating a risk for social isolation.

Frailty was assessed using the Canadian Study of Health and Aging (CSHA) Clinical Frailty Scale (34) evaluated by the interviewer at the end of the interview with a seven-point response scale ranging from 1 = *very fit* to 7 = *very frail*. Furthermore, patients’ GPs completed questionnaires about the presence of comorbidities at each study wave. A comorbidity score was calculated by adding the number of comorbidities.

### Statistical Analyses

All statistical analyses were performed using SPSS 24 for Windows (SPSS Inc., Chicago, IL, USA). For all analyses, an alpha level of less than 0.05 was considered statistically significant. Descriptive data are presented as means ± standard deviations or case numbers and percentages as appropriate. In order to investigate the association between anxiety and possible predictors, we divided patients into two groups according to their GAI-SF score: patients with anxiety and patients without anxiety. Patients were assigned to the anxiety group when ratings were ≥3 on the GAI-SF. A cutoff of ≥3 for the GAI-SF was suggested by Johnco et al. (27) with a sensitivity of 78.14% and a specificity of 98.3% for detecting generalized anxiety disorder (GAD). Patients with less than three points on the GAI-SF were assigned to the no anxiety group. Differences of baseline characteristics between subjects with anxiety and without anxiety were analyzed using χ^2^ test for categorical variables or Mann–Whitney *U* test for continuous variables. A binary logistic regression model was developed to identify risk factors predictive of anxiety symptoms in the oldest-old. Anxiety was used as the dependent variable. Independent variables simultaneously included in the regression model were recent experience of loss, sociodemographic variables (age, sex, and educational level), presence of depression, frailty, physical illness, cognitive status, and social isolation. Adjusted odds ratios (OR) and 95% confidence intervals (95% CI) are stated. The relationship between anxiety and loss was further analyzed separately for patients with depressive symptoms and patients without depressive symptoms using χ^2^ test for categorical variables.

## Results

### Characteristics of the Sample

**Table 1** displays the characteristics of the study sample. The mean age of the sample was 86.8 (SD = 3.02). More than half of the study sample was female (N = 589, 65.7%), was widowed (N = 513, 57.2%), and had a low educational level (N = 514, 57.3%). Excluded individuals were more frequently female [74.2% vs. 65.7%; χ^2^(1,1342) = 9.942, p < .01], had a lower educational level [χ^2^(1,1342) = 11.730, p < .01], but did not differ with regard to age (M = 86.8, SD = 3.4 vs. M = 86.7, SD = 3.02; p = .670). Out of the 897 GP patients that were included in the analysis, 767 (85.5%) had no anxiety, while 130 (14.5%, 95% CI: 12.4–16.8) had anxiety according to GAI-SF ≥3. No significant differences were found between the subsamples of older patients with and without anxiety regarding age, gender, marital status, and educational attainment. Compared to individuals without anxiety, the group with anxiety had a significantly higher rate of experiencing a recent loss in the 18 months prior to the assessment (p < .05), had a lower MMSE score (26.9 vs. 27.7), were more frequently depressed (45.4% vs. 7.6%), were more frail (CSHA mean score: 4.1 vs. 3.5), had more physical illnesses (mean score: 6.5 vs. 5.9), and had a lower score on the LSNS-6 (12.2 vs. 14.2). In the total sample, 30.5% reported a recent loss; among those without anxiety, 29.1% had a recent loss, while 39.2% of the subsample with anxiety experienced a recent loss. People close to the patients (friends, neighbors) other than relatives account for the highest proportion among people lost (49.3% in the no anxiety group, 52.9% in the anxiety group). Loss of a child (2.2% in the no anxiety group) and loss of a spouse (9% in the no anxiety group, 9.8% in the anxiety group) were the most uncommon types of loss reported.

**Table 1:** Sociodemographic characteristics of the patient sample at follow-up wave 5.

	Total sample (n = 897)	No anxiety, GAI-SF < 3 (n = 767, 85.5%)	Anxiety, GAI-SF ≥ 3 (n = 130, 14.5%)	p^a^
GAI, mean ± SD	1.08 ± 1.30	0.65 ± 0.77	3.6 ± 0.75	*<.001*
Age, years				
Mean ± SD	86.8 ± 3.02	86.8 ± 3.1	86.5 ± 3.05	.265
Range	82–98	82–98	82–96	
Gender, n (%)				
Male	308 (34.3)	270 (35.2)	38 (29.2)	.195
Female	589 (65.7)	497 (64.8)	92 (70.8)	
Education^b^, n (%)				
High	130 (14.5)	118 (15.4)	12 (9.2)	.184
Middle	253 (28.2)	214 (27.9)	39 (30.0)	
Low	514 (57.3)	435 (56.7)	79 (60.8)	
Marital status, n (%)				
Single/divorced	104 (11.6)	82 (10.7)	22 (16.9)	.114
Married	280 (31.2)	240 (31.3)	40 (30.8)	
Widowed	513 (57.2)	445 (58.0)	68 (52.3)	
MMSE, mean ± SD	27.5 (2.2)	27.7 (2.1)	26.9 (2.7)	*.003*
Depressive symptoms^c^, n (%)	117 (13.0)	58 (7.6)	59 (45.4)	*<.001*
CSHA, mean ± SD	3.6 (1.5)	3.5 (1.5)	4.1 (1.5)	*<.001*
LSNS, mean ± SD	13.9 (5.6)	14.2 (5.4)	12.2 (6.3)	*<.001*
Number of physical illnesses, mean ± SD	6.0 (3.1)	5.9 (3.0)	6.5 (3.2)	*.044*
Experience of loss since last assessment, n (%)	274 (30.5)	223 (29.1)	51 (39.2)	*.023*

GAI-SF, short form of Geriatric Anxiety Inventory; SD, standard deviation; Values in italics indicate statistical significance.

a Variables analyzed based on *χ* ^2^ tests or nonparametric Mann–Whitney U tests, as appropriate.

b Classification according to the international new CASMIN educational classification (25): Low, inadequately completed general education, general elementary education, basic vocational qualification, or general elementary education and vocational qualification; Middle, intermediate vocational qualification or intermediate general qualification and vocational qualification, intermediate general qualification, general maturity certificate, vocational maturity certificate/general maturity certificate, and vocational qualification; High, lower tertiary education—general diplomas/diplomas with vocational emphasis, higher tertiary education—lower level/higher level; MMSE, Mini-Mental State Examination.

c Based on the Geriatric Depression Scale; CSHA, Canadian Study of Health and Aging Clinical Frailty Scale; LSNS, Lubben Social Network Scale.

**Table 2** displays the prevalence of anxiety symptoms for three different age groups (82–85, 86–90, and >90 years) and gender. Compared to the age group of 82–85 year olds, the group of patients between 86 and 90 years had a slightly higher prevalence of anxiety symptoms (15.9%, 95% CI: 12.6–19.2 vs. 14.6%, 95% CI: 11.4–18.5). However, the lowest prevalence of anxiety symptoms was found in the oldest age group (>90 years) with 8.4% (95% CI: 3.5–14.2).

**Table 2:** Age- and gender-specific prevalence of anxiety symptoms.

	82–85 years, n = 362			86–90 years, n = 428			>90 years, n = 107			Total sample (82+)		
	Male	Female	Total	Male	Female	Total	Male	Female	Total	Male	Female	Total
	n = 15	n = 38	n = 53	n = 21	n = 47	n = 68	n = 2	n = 7	n = 9	n = 38	n = 92	n = 130
GAI-SF ≥ 3, %	10.6	17.2	14.6	15.6	16.0	15.9	6.3	9.3	8.4	12.3	15.6	14.5
95% CI	6.0–15.5	12.6–22.1	11.4–18.5	9.4–21.9	12.0–20.4	12.6–19.2	0–16.7	3.7–16.4	3.5–14.2	8.8–16.2	12.9–18.6	12.4–16.8

GAI-SF, short form of the Geriatric Anxiety Inventory; CI, confidence interval.

### Associated Factors

In **Table 3**, the results of the cross-sectional binary logistic regression are shown. The χ^2^ difference between the null model and the model containing the predictors was significant [χ^2^(11) = 124.8] at the p < .01 level. Variables found to be significantly associated with anxiety were experience of a preceding loss, depressive symptoms, and cognitive ability. The odds of reporting anxiety according to GAI-SF ≥3 was 1.91 (95% CI 1.15–3.17) times larger for those patients who experienced a loss in the 18 months prior to the assessment. Furthermore, the results showed that older individuals with depressive symptoms had 9.26 (95% CI 5.1–16.87) times the odds of reporting anxiety symptoms compared to those individuals without depressive symptoms. In addition, the MMSE score was found to be associated with the presence of anxiety symptoms: higher MMSE scores were associated with a significantly lower chance of reporting anxiety (OR 0.89, 95% CI 0.81–0.97). Results of the regression analysis showed no significant association between anxiety and gender, age, educational level, frailty, social network, and physical illness. Furthermore, the interaction between depression and loss was not significantly associated with anxiety.

**Table 3:** Results of the logistic regression analysis for symptoms of anxiety.

	ß (S.E.)	OR (95% CI)	Wald	p-value
Gender				
Male	0.047 (0.24)	1.05 (0.66–1.67)	0.04	0.845
Age	−0.043 (0.04)	0.96 (0.89–1.03)	1.44	0.230
Education				
High	Ref.			
Middle	0.49 (0.39)	1.65 (0.77–3.53)	1.63	0.201
Low	0.38 (0.37)	1.47 (0.72–3.00)	1.11	0.293
Recent loss	0.65 (0.26)	1.91 (1.15–3.17)	6.29	0.012
Depressive symptoms^a^				
No	Ref.			
Yes	2.23 (0.30)	9.26 (5.1–16.87)	52.8	0.000
CSHA	−0.01 (0.08)	0.99 (0.84–1.16)	0.02	0.890
MMSE	−0.12 (0.05)	0.89 (0.81–0.97)	6.36	0.012
LSNS	−0.03 (0.02)	0.97 (0.94–1.01)	1.87	0.171
Physical illness	0.03 (0.04)	1.03 (0.96–1.11)	0.79	0.372
interaction depressive symptoms*loss	−0.04 (0.49)	0.96 (0.37–2.51)	0.01	0.930

R2 = .130 (Cox & Snell), .231 (Nagelkerke). Model χ^2^(11) = 124.804, p < .01.

S.E., standard error; OR, odds ratio; CI, confidence interval; Ref., reference category.

a Based on the Geriatric Depression Scale; CSHA, Canadian Study of Health and Aging Clinical Frailty Scale; MMSE, Mini-Mental State Examination; LSNS, Lubben Social Network Scale.

**Table 4** shows the univariate analysis of the association between loss and anxiety symptoms separated for patients with and without depressive symptoms. A significant difference was found for the subsample of patients without depressive symptoms between those patients with and without anxiety regarding the experience of a recent loss. Among those patients without depressive symptoms, a significantly higher proportion of older people with anxiety also reported a recent loss compared to those in the no-anxiety group (42.3% vs. 29.3%).

**Table 4:** Experience of loss according to depression and anxiety measures.

	Patients without depressive symptoms^a^, n = 780			Patients with depressive symptoms^a^, n = 117		
	No anxiety GAI-SF < 3	Anxiety GAI-SF ≥ 3	p^b^	No anxiety GAI-SF < 3	Anxiety GAI-SF ≥ 3	p^b^
Experience of loss since last assessment, n (%)	208 (29.3)	30 (42.3%)	*.030*	15(25.9)	21(35.6)	.318

a Classification according to the Geriatric Depression Scale using a cutoff score of ≥6 to indicate a depressive symptomatology; GAI-SF, short form of the Geriatric Anxiety Inventory.

b Variables analyzed based on *χ* ^2^ tests. Values in italics indicate statistical significance.

## Discussion

The present study provides results on the age- and gender-specific prevalence of anxiety and its associated variables in a sample of oldest-old primary care patients. We found 14.5% (95% CI: 12.4–16.8) of community-dwelling people aged 82 years and older experiencing anxiety according to GAI-SF ≥3. Older patients who experienced a loss in their closer social environment within the last 18 months had almost two times the odds (OR: 1.91) of reporting anxiety compared to those without a recent loss. Similarly, depression and cognitive status were associated with anxiety. No relation was found between social network, gender, age, frailty, or physical illness and anxiety in regression analysis.

### Prevalence of Anxiety in Late Life

A point prevalence of 14% for anxiety in the oldest-old is comparable to results found in “younger” people in old age (e.g., 55+ or 65+ years). Anxiety disorders have been found to be present in 14–17% of older adults (3, 35, 36). Kirmizioglu et al. report a current prevalence for any anxiety disorder of 17.1% in a sample of older adults (37). In individuals aged 80 years or older, the authors found a prevalence of 4% for current GAD and social phobia and a prevalence of 10% for current specific phobia (37). Similarly, a multicenter study found a 12-month prevalence rate of 17.2% for one or more anxiety disorders in a large population study of older adults aged 65–84 years (3). However, with regard to age differences, they found a drop in the overall prevalence of 47% in the age group of 80–84 year olds compared with younger age groups (65–69 years) (3). In contrast, somewhat higher proportions have been reported by Forlani et al. in a sample of community-dwelling older people aged 74 years and older (28). The authors reported a point prevalence of anxiety symptoms of 21% measured with the GAI-SF (28).

Measuring anxiety in old age is associated with more barriers than in younger age groups. For instance, a higher likelihood of physical problems and illnesses may complicate the diagnosis of an anxiety disorder (38). Furthermore, older adults have been reported to present anxiety often as physical symptoms or otherwise minimize their worries when specifically asked about it (28). Due to diagnostic challenges, anxiety disorders may therefore be underdiagnosed and undertreated (39–41). Anxiety measures typically used with younger adults have been criticized for the use with older people for being too long, including reversed scored items and relying too much on somatic symptom reporting (42). In contrast, the GAI-SF Inventory offers an efficient way to measure anxiety symptoms in older adults while avoiding typical problems of other anxiety measures as too many items or a too complicated response format. Furthermore, the GAI-SF has been shown to have good psychometric properties (26, 27) and can therefore be perceived as a valid instrument to assess anxiety in old age.

### Experience of Loss as a Risk Factor for Anxiety in Late Life

Experiencing the loss of a family member or a close friend is a frequent negative life event in later life (43) and has been associated with an increased stress response (44) and adverse health effects (14, 45–47) in older adults. Bereavement therefore necessitates extensive processes of adjustment including grief, reorientation, and strengthening other relationships. This may be particularly challenging with decreasing social networks and diminishing social support in later life. Furthermore, the death of a close person may remind older people of their own mortality and has previously been found to be associated with depression (12, 45). Bereavement and experience of loss in old age have been predominantly analyzed with regard to depressive symptoms (12, 45, 48) or psychological well-being in general (49), while the association of bereavement and anxiety in old age is less well understood. In a systematic review on the prevalence of mood and anxiety disorders following the loss of a spouse (14), only 1 out of 11 studies provided results on anxiety disorders. In this study, the authors found elevated prevalence rates for panic disorder and GAD in widowed individuals 6 months after the loss compared to the community prevalence rate of those disorders in the same area (50). In line with previous findings, our results support the notion that recent experience of loss in older adults should be taken seriously in the context of clinical practice as an independent risk factor for anxiety in late life.

Most studies investigating the effects of bereavement on psychological morbidity in late life solely considered widowhood and therefore the loss of a spouse (48, 51). However, this may not truly represent loss experiences of older adults as they experience more frequent cases of death in their close social environment including friends, siblings, children, or other family members. An advantage of this study is its comprehensive conceptualization of loss considering a broad range of loss experiences rather than just the loss of a spouse. The results showed that experiencing the loss of any kind of close friend or relative was found to be a significant predictor of anxiety symptoms in the oldest-old.

Apart from anxiety and depression, posttraumatic stress disorder (PTSD) has been found to be a frequent reaction following the experience of loss (14, 52, 53). With regard to anxiety, PTSD has been reported to share some common symptoms (e.g., hyperarousal) and has been shown to be highly comorbid with several anxiety disorders (54). Consequently, PTSD (e.g., as a reaction to the experience of loss) may be a potential confounder mediating the relationship between experience of loss and anxiety symptoms. However, PTSD is notably characterized by the situation-specific nature of its symptoms and predominant rumination about past events (55). On the other hand, the GAI-SF, which we used here to assess anxiety, focuses primarily on symptoms of more widespread worrying [e.g., “Little things bother me a lot” and “I think of myself as a worrier” (26)]. Still, as PTSD has not been assessed in this study, the possibility of PTSD as a confounder cannot be ruled out completely. Therefore, future studies should continue to investigate the relation of loss and anxiety in late life including PTSD and considering previous psychiatric conditions throughout a lifetime.

Depressive symptoms and even anxiety are perceived as a normal process after bereavement (14). Still, in the oldest-old, there may also be the risk of underdiagnosing a real mental health problem with concomitant stresses and functional limitations (35).

### Depression as a Potential Confounder

Anxiety and depression often co-occur in life (56, 57). Similarly, we found depression significantly associated with late-life anxiety in cross-sectional analyses. While the GAI-SF has shown good psychometric properties with regard to internal consistency and convergent validity, it has also been found to correlate with the GDS—a measure of depression (27). There is, however, no agreement on the interpretation of this correlation in the literature. On the one hand, the correlation between the GAI-SF and the GDS is seen as weak divergent validity (27, 58); on the other hand, it has been interpreted as evidence for convergent validity because of the high co-occurrence of anxiety with depression (59). Poor divergent validity with regard to measures of depression is a problem shared by other anxiety measures (60) and has been discussed as the result of a general concept similarity of both anxiety and depression (27). With regard to the relationship between loss and anxiety, no interaction effect for loss and depression was found in the current study, indicating experience of loss as a potential independent risk factor for late-life anxiety. Furthermore, in the subsample of patients without depressive symptoms, a significantly higher proportion of older people with anxiety reported the experience of a recent loss compared to those without anxiety. The results of our study therefore point to a relationship between experience of loss and late-life anxiety independent of depression. Future studies should further investigate this relationship with regard to specific anxiety disorders and including professional diagnosis.

### Further Correlates of Anxiety in Late Life

Aside from depression and loss as putative risk factors for anxiety symptoms in late life, we found cognitive status significantly associated with anxiety in the oldest-old. In line with our results, previous studies found impairments in memory and further neuropsychological domains (e.g., problem solving or information processing speed) associated with anxiety in older adults (61, 62). While various theories have been proposed with regard to the direction and impact of this relationship between anxiety and cognitive impairment (63–66), the cross-sectional results of this study indicate that the association of anxiety and diminished cognitive status applies even for the oldest-old. In contrast with previous literature (22, 28, 36, 61), female sex as well as physical illness or frailty were not associated with anxiety in regression analysis in our sample of community-dwelling people aged 82 years and older. Previous studies analyzed anxiety in considerably smaller and younger samples using different anxiety measures (e.g., Hospital Anxiety and Depression Scale or Hamilton Anxiety Rating Scale) compared to our study. A reason for our null results with regard to female sex, physical burden, and anxiety in this study might further be explained by a healthy survivor effect in the oldest-old with diminishing effects of classical risk factors for anxiety typically found in younger age groups. In line with our results, Schuurmans et al. (67) could not find sociodemographic variables or physical health and chronic diseases as potential risk factors of persistent anxiety in older adults. According to the authors, the results indicate an unfavorable long-term outcome of anxiety in older adults with neuroticism as a strong prognostic factor (67). However, with regard to sex, we found higher prevalence rates of anxiety symptoms in older women compared to older men. A further reason for our null results with regard to sex in the regression analysis might therefore be explained by the inclusion of variables mediating this gender effect (e.g., depressive symptoms).

### Strengths and Limitations

To the best of our knowledge, this is the first study investigating the relationship between recent experience of loss, as well as further covariates and anxiety symptoms in a sample of oldest-old community-dwelling GP patients. A major strength of this study is its focus on the very old and its inclusion of adults aged 82 years and older. The data are based on a large cohort of older GP patients assessed in several centers across Germany, thereby increasing the representative level of the sample. Almost all German seniors see their GP on a regular basis. Primary care serves as a major access point to the first level of care in older adults, thus providing a key setting for the recruitment with high representative nature. The assessment of anxiety symptoms was based on the GAI-SF, a valid instrument specifically developed to assess anxiety in old age (26, 27). A further strength of this study is its comprehensive conceptualization of loss considering a broad range of loss experience. Also, several limitations of the present study have to be addressed. First, the generalizability of our results may be limited due to a selection bias as a number of GP patients refused study participation or had to be excluded from analysis because of missing information. Second, anxiety symptoms were assessed using a validated screening tool and not by a clinical interview or professional diagnosis according to the International statistical classification of diseases and related health problems, 10th revision (ICD-10) or Diagnostic and statistical manual of mental disorders, 5th revision (DSM-5). This screening tool for anxiety in old age does not allow for drawing conclusions on different kinds of anxiety disorders and may be associated with some inaccuracy. Furthermore, psychiatric conditions throughout a lifetime (e.g., as PTSD, previous anxiety disorders) have not been assessed during the clinical interviews and could be potential confounding variables. Similarly, psychotropic drugs have not been included in the analyses and may be a potential confounding variable. Finally, due to the cross-sectional design of this study, conclusions about the causality of the relationship between the investigated variables cannot be drawn.

## Conclusion

Anxiety symptoms are a frequent mental health problem in latest life. Our findings support the notion that depression, slightly impaired cognitive ability, and recent experience of loss are significantly associated with anxiety in the oldest-old and constitute an important burden for mental health in late life. Our findings further support the idea that recent experience of loss should be taken seriously in the context of clinical practice with respect to diagnosing and treating anxiety in old age. Future studies should extend our results and analyze the relationship between loss and anxiety in late life with regard to specific anxiety disorders using standardized diagnostic instruments such as the Composite International Diagnostic Interview (CIDI) or the Structured Clinical Interview for DSM-IV (SCID). Moreover, further research is needed to investigate the course of anxiety symptoms in late life using a longitudinal approach.

## Members of the AgeCoDe and AgeQualiDe Study Group

Principal Investigators*: Wolfgang Maier, Martin Scherer, Steffi G. Riedel-Heller, Heinz-Harald Abholz, Christian Brettschneider, Cadja Bachmann, Horst Bickel, Wolfgang Blank, Hendrik van den Bussche, Sandra Eifflaender-Gorfer, Marion Eisele, Annette Ernst, Angela Fuchs, André Hajek, Kathrin Heser, Frank Jessen, Hanna Kaduszkiewicz, Teresa Kaufeler, Mirjam Köhler, Hans-Helmut König, Alexander Koppara, Diana Lubisch, Tobias Luck, Dagmar Lühmann, Melanie Luppa, Tina Mallon, Manfred Mayer, Edelgard Mösch, Michael Pentzek, Jana Prokein, Alfredo Ramirez, Susanne Röhr, Anna Schumacher, Janine Stein, Susanne Steinmann, Franziska Tebarth, Carolin van der Leeden, Michael Wagner, Klaus Weckbecker, Dagmar Weeg, Jochen Werle, Siegfried Weyerer, Birgitt Wiese, Steffen Wolfsgruber, Thomas Zimmermann. *Hendrik van den Bussche (2002–2011).

## Ethics Statement

The study was conducted in accordance with the Declaration of Helsinki and was approved by the local ethic committees of all participating centers: Ethics Commission of the Medical Association Hamburg, Ethics Commission of the University of Bonn, Medical Ethics Commission II, University of Heidelberg at the University Medical Center of Mannheim, Ethics Commission at the Medical Center of the University of Leipzig, Ethics Commission of the Medical Faculty of the Heinrich-Heine-University Düsseldorf, and Ethics Committee of the TUM School of Medicine, Munich. All patients and/or their proxies provided written informed consent.

## Author Contributions

SRH, BW, HB, MP, HHK, MS, WM, and SW contributed to the conception and design of the study. SR, BW, CVDL, KH, JW, AF, and JS contributed to the acquisition of data. FW performed the analysis of data. FW, JS, and SRH conducted the interpretation of data. Drafting of the article was done by FW, JS, and SRH. SRH, JS, SR, BW, CVDL, KH, HB, MP, HHK, JW, AF, MS, WM, SW, EM, AO, AH, LK, HVDB, and MW revised the manuscript critically for important content. All authors read and approved the final manuscript.

## Funding

This publication is part of the German Research Network on Dementia (KND), the German Research Network on Degenerative Dementia (KNDD; German Study on Ageing, Cognition and Dementia in Primary Care Patients; AgeCoDe), and the Health Service Research Initiative [study on Needs, health service use, costs and health-related quality of life in a large sample of oldest-old primary care patients (85+; AgeQualiDe)] and was funded by the German Federal Ministry of Education and Research (grants KND: 01GI0102, 01GI0420, 01GI0422, 01GI0423, 01GI0429, 01GI0431, 01GI0433, 01GI0434; grants KNDD: 01GI0710, 01GI0711, 01GI0712, 01GI0713, 01GI0714, 01GI0715, 01GI0716; grants Health Service Research Initiative: 01GY1322A, 01GY1322B, 01GY1322C, 01GY1322D, 01GY1322E, 01GY1322F, 01GY1322G). Furthermore, this publication is part of the study “Healthy Aging: Gender-specific trajectories into latest life” (AgeDifferent.de) and the study “Improving care of late-life depression: Acceptability, effectiveness and cost-effectiveness of the web-based self-management E-couch “Bereavement and Loss” program” (AgE-health) and was funded by the German Federal Ministry of Education and Research (AgeDifferent.de: Funding program “Gesund – ein Leben lang,” grants 01GL1714A; 01GL1714B; 01GL1714C; 01GL1714D; AgE-health: 01GY1613). We want to thank all participating patients and their general practitioners for their good collaboration.

## Conflict of Interest Statetement

The authors declare that the research was conducted in the absence of any commercial or financial relationships that could be construed as a potential conflict of interest.
